# Real-time single-molecule studies of the motions of DNA polymerase fingers illuminate DNA synthesis mechanisms

**DOI:** 10.1093/nar/gkv547

**Published:** 2015-05-26

**Authors:** Geraint W. Evans, Johannes Hohlbein, Timothy Craggs, Louise Aigrain, Achillefs N. Kapanidis

**Affiliations:** Department of Physics and Biological Physics Research Group, Clarendon Laboratory, University of Oxford, Parks Road, Oxford OX1 3PU, United Kingdom

## Abstract

DNA polymerases maintain genomic integrity by copying DNA with high fidelity. A conformational change important for fidelity is the motion of the polymerase fingers subdomain from an open to a closed conformation upon binding of a complementary nucleotide. We previously employed intra-protein single-molecule FRET on diffusing molecules to observe fingers conformations in polymerase–DNA complexes. Here, we used the same FRET ruler on surface-immobilized complexes to observe fingers-opening and closing of individual polymerase molecules in real time. Our results revealed the presence of intrinsic dynamics in the binary complex, characterized by slow fingers-closing and fast fingers-opening. When binary complexes were incubated with increasing concentrations of complementary nucleotide, the fingers-closing rate increased, strongly supporting an induced-fit model for nucleotide recognition. Meanwhile, the opening rate in ternary complexes with complementary nucleotide was 6 s^−1^, much slower than either fingers closing or the rate-limiting step in the forward direction; this rate balance ensures that, after nucleotide binding and fingers-closing, nucleotide incorporation is overwhelmingly likely to occur. Our results for ternary complexes with a non-complementary dNTP confirmed the presence of a state corresponding to partially closed fingers and suggested a radically different rate balance regarding fingers transitions, which allows polymerase to achieve high fidelity.

## INTRODUCTION

DNA polymerases maintain genomic integrity by replicating and repairing cellular DNA with high fidelity (1). For example, *Escherichia coli* DNA polymerase I incorporates incorrect nucleotides only once every 10 000–100 000 bases (2), despite their structural similarity to the correct, complementary deoxyribonucleoside triphosphates (dNTPs). DNA polymerases achieve this accuracy for a range of substrates, altering their nucleotide selectivity based on the DNA templating base.

**Figure 1.**
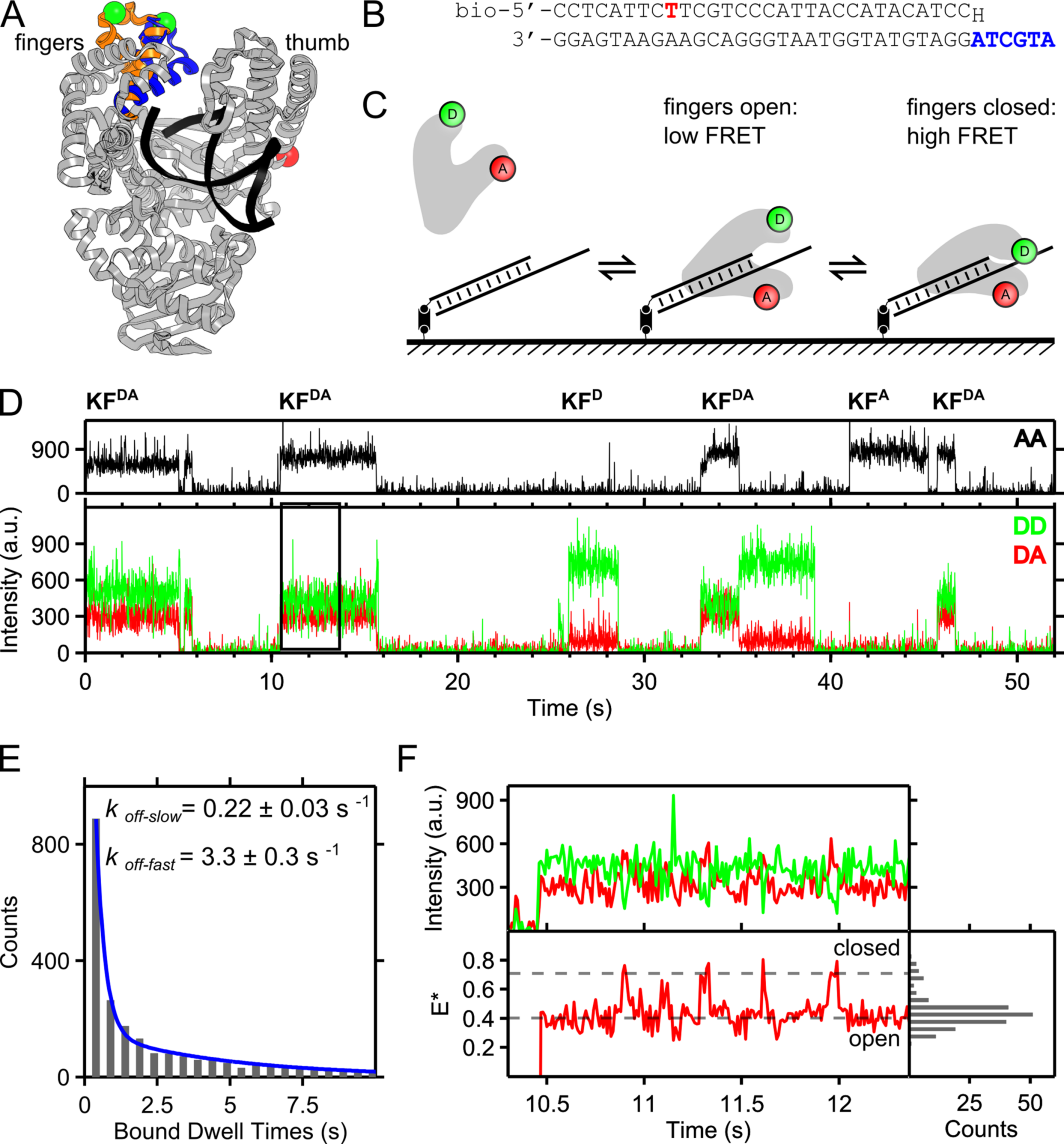
An intra-protein FRET assay for real-time monitoring of fingers dynamics. (**A**) The fingers-closing conformational change as inferred from crystal structures of *B. Stearothermophilus* (*Bst*) DNA Polymerase (5), a close homologue of Klenow Fragment. The crystal structures of Pol-DNA binary complex (PDB 1L3U) and the Pol–DNA–dCTP ternary complex (PDB 1LV5) represent different positions of the mobile O-helix of the fingers subdomain, shown in orange for the open conformation (1L3U) and blue for the closed conformation (1LV5), respectively. The protein backbone is shown in gray and the DNA strands in black (both shown for 1L3U). The labeling positions are indicated, with C_β_ atoms shown in green (Cy3B as the donor) on fingers and red (ATTO647N as the acceptor) on the thumb. (**B**) Base-paired primer–template oligonucleotide used in our studies. In the primer DNA, position −22 is labeled with a red fluorophore, the 5′ terminal is biotinylated for surface immobilization, and the 3′ terminal base is dideoxy-modified (H) to prevent primer extension. The single-stranded overhang of the templating DNA is shown in blue. (**C**) After photobleaching the red fluorophore attached to the DNA, transient binding of KF molecules to the DNA is monitored and the conformational changes within KF are characterized using the internal FRET pair. (**D**) Fluorescence emission as a function of time at a localized DNA molecule. Upper panel: Acceptor fluorescence upon direct acceptor excitation (AA; black trace). Lower panel: donor (DD) and acceptor (DA) fluorescence upon donor excitation. Sudden increases and decreases of the fluorescence intensity are assigned to polymerase binding and dissociation events. Comparing the three traces, we identify the labeling stoichiometry of associated KF molecules (KF superscripted with D (donor) and/or A (acceptor)). Data collected using 10 ms ALEX; 3 mW green and 1.5 mW red excitation; 4 nM Pol I (KF) and 0.125 μM dTTP (complementary nucleotide). The black box (encompassing the segment of 11–14 s) is expanded in (F). (**E**) KF-DNA complex dwell times reveal bimodal dissociation kinetics. Binding events refer to KF^D^ and KF^DA^ proteins. Fast and slow dissociation rates are assigned to KF–surface and KF–DNA binding respectively (see Supplementary Figure S1). Long frame time (100 ms) and low laser intensity (0.3 mW green) are used to increase fluorophore photobleaching lifetime. (**F**) Expanded binding event in (D) with fluorescence trace (upper panel) and corresponding FRET proximity ratio (E* lower panel). Interconversions between FRET values at E* ∼0.4 and E* ∼0.6 are seen, coincident with anti-correlated DD and DA signals (AA signal remains constant; not shown). A histogram of E* values for each frame is shown, with the polymerase predominantly in the low-FRET state.

Fidelity mainly depends on pre-chemistry reaction steps that help discriminate and reject incorrect nucleotides before they are incorporated in DNA (3). A large conformational change important for fidelity is the motion of the polymerase ‘fingers’ sub-domain, which moves from an open conformation (as seen in the polymerase–DNA binary complex) to a closed conformation upon binding of a complementary nucleotide (Figure 1A) (4–7). To directly monitor fingers-closing in DNA Polymerase I (Klenow fragment, KF), we previously used a single-molecule FRET strategy based on confocal microscopy on diffusing molecules. Our work showed that the KF fingers have different conformational profiles depending on the presence of nucleotides and DNA templates (8) (Figure 1A). The KF–DNA binary complex was found mainly in the fingers-open conformation, but also occupied a fingers-closed conformation. On the other hand, the KF–DNA–(A-dTTP) ternary complex (with a complementary nucleotide) was found mainly in the fingers-closed conformation, but also explored a fingers-open conformation. The presence of minor states raised the possibility that both binary and ternary complexes are dynamic, and populate the minor state transiently. In the presence of incorrect substrates (non-complementary dNTPs or complementary rNTPs), ternary complexes assumed a third, partially closed, conformation. We extended our conformational analysis to mutator DNA polymerases (9) and showed that mutator phenotypes were linked to weaker binding to complementary dNTP and increased tendency to form partially closed conformations; effectively, complementary nucleotides were treated similarly to non-complementary nucleotides by these mutator polymerases. We also inferred the rates of interconversion between conformations by analyzing the width and shape of FRET distributions for binary and ternary complexes, under the assumption of a dynamic equilibrium between states (8–11).

Our single-molecule FRET studies highlighted the importance of fingers-closing for fidelity; however, due to the brief molecular dwell (1–2 ms) within the confocal detection volume, we could not study directly the kinetics of fingers-closing and opening in KF-DNA complexes. As a result, it has remained elusive whether there is a dynamic equilibrium between major and minor states (instead of an ensemble of static, non-interconverting populations). Further, since the rates of any transitions have not been directly determined, they could not be compared to the kinetics of the reaction rate-limiting step.

To measure the kinetics of the conformational changes of the fingers subdomain, extended observations of surface-immobilized polymerase complexes are needed; such a capability will also help monitor the fingers subdomain during DNA polymerization. Previous single-molecule work inferred fingers-closing in T7 DNA polymerase via fluorescence-intensity changes of an environmentally sensitive dye (12); however, the structural origin of such changes was difficult to ascertain, and the extracted rates did not correspond to fingers-closing rates measured using bulk fluorescence (12–14). Additionally, surface-immobilization in conjunction with single-molecule FRET has previously allowed extended observations of protein conformations (15,16). However, structural interconversion rates were not extracted, nor was it possible to deconvolve unequivocally any protein structural changes from DNA rearrangements or potential translocation events, as these assays relied on FRET between a singly labeled polymerase and labeled DNA.

Here, we use single-molecule FRET within doubly labeled KF bound to surface-immobilized DNA molecules to visualize and characterize KF fingers-opening and closing (Figure 1C). Our high temporal resolution (10 ms) and long observation spans allowed us to directly observe the fast dynamics of the fingers in the binary complex and to measure the structural interconversion rates. We characterized the kinetics of fingers-closing and fingers-opening with complementary nucleotide and linked it to KF reaction mechanisms and fidelity. The characterization of both binary and ternary complex dynamics shows that fingers-closing in the presence of complementary nucleotide occurs via an induced-fit mechanism. Finally, our results on complexes with non-complementary dNTPs confirmed the presence of a FRET state that correspond to a partially closed state of the fingers, which serves as a fidelity checkpoint (3,9,15).

## MATERIALS AND METHODS

### Protein and DNA preparation

KF labeled with Cy3B and ATTO647N on the 744 (fingers) and 550 (thumb) residues was prepared as described previously (9). Primer–template oligonucleotides were synthesized, dye-labeled and HPLC-purified by IBA Life Sciences (Germany) with sequences shown in Figure 1B. The DNA primer strand was 5′ biotinylated and 3′ dideoxy modified, and labeled with Cy5 at the −22 position. The dideoxy termination confined the analysis to reaction steps occurring before the chemical step, and may influence steps such as metal binding in the ternary complex.

### TIRF experiments

Passivated glass surfaces were produced by burning glass coverslips (25 × 65 mm, thickness number 1, VWR) in a furnace in which the cover slips were heated at a rate 90°C/h, held at 500°C for 1 h, and cooled at 50°C/h. Burned glass surfaces were then washed in acetone for 5 min, and incubated in a 1:50 Vectabond (Vector Labs) to acetone solution, for 5 min. The slides were then washed with MilliQ water and dried using compressed nitrogen. Silicone gaskets (Grace Bio-labs) were placed on the dried slides, and each well was incubated with 20 μl of 200 mg/ml NHS-PEG (PEG-SVA MW 5000 Da, Laysan Bio) 2.5 mg/ml NHS-biotin-PEG (MW 5000 Da, Laysan Bio) in 50 mM MOPS at pH 7.5. After 3 h, the wells were washed and stored in phosphate buffered saline (PBS) buffer. DNA molecules were immobilized to the passivated glass slides by incubating with 0.02 mg/ml neutravidin for 1 min, followed by incubation with 10–100 pM biotinylated primer–template DNA for 1 min.

Surface-immobilized DNA molecules were imaged using a custom-built TIRF microscope (17), in a solution consisting of a variable KF concentration in 50 mM Tris, pH 7.5, 100 μg/ml bovine serum albumin (BSA), 1 mM dithiothreitol (DTT), 5% glycerol, 10mM MgCl_2_, 1 mM Trolox (UV-treated (18)), 1.9 mg/ml glucose oxidase, 0.075 mg/ml catalase, and 1.5% (w/v) d-glucose. This solution was sealed in the silicone gasket using a glass coverslip. When alternating laser excitation (ALEX) was used during imaging, the 532- and 640-nm lasers were alternated every frame, such that successive frames collected data from fluorescence emission under donor and then acceptor excitation respectively. Ultrapure nucleotides were purchased from Sigma-Aldrich.

### Binary and ternary complex experiments

To investigate the dynamics of the fingers of KF in the presence or absence of nucleotide, DNAs were immobilized on a passivated glass surface in a solution containing 4 nM KF and a range of dTTP concentrations (dTTP is complementary to the first templating base). Data was acquired for 20 min using continuous green excitation at 1.5 mW, with a 10 ms frame time. Data from 96, 100, 78, 100, 89, 78 and DNA molecules were collected for nucleotide concentrations of 0, 0.1, 0.3, 1, 3 and 10 μM dTTP, respectively.

### Intensity trace extraction

Movies of surface-immobilized DNA molecules with KF transiently binding from solution were analyzed using our twoTone software. (19). We obtained the positon of the DNA molecules on the surface using the fluorescence signal from the attached dye. Fluorescence intensity traces were then obtained by fitting a Gaussian profile at these locations, in all subsequent frames. This generated a fluorescence time-trace for each DNA molecule, which described the association and dissociation of fluorescently labeled KF to the DNA. When the position of the KF binding event was required (Supplementary Figures S1 and S2), the binding events were localized by allowing the Gaussian profiles to freely fit their X/Y locations within 1 pixel of the initially localized position.

To identify KF binding events from a fluorescence time-trace, we used a low intensity threshold (300–500 a.u.), ignoring any single frame changes. A further threshold was then applied, requiring the mean intensity at each KF binding event to be 1.5× the initial intensity threshold. This allowed the extraction of continuous KF binding events, even if there was a transient dip in fluorescence due to changes in background intensity. The apparent FRET efficiency was calculated using the donor and acceptor fluorescence intensities under donor excitation (DD/DA respectively) according to the equation ${E^*} = {\rm DA/(DA} + {\rm DD)}$.

### Hidden Markov modeling

Hidden Markov modeling (HMM) was performed to extract dwells in KF's open and closed fingers conformations, using previously documented software (20), which was based on the vbFRET package. Hidden Markov modeling was performed on the concatenated FRET traces of KF–DNA complexes at each DNA separately, fit to 1 to 5 state HMM models, with the appropriate number of states selected using maximum evidence criteria (20,21). The five possible states represented the FRET contributions from: two fingers-open states (E* ∼ 0.4); two fingers-closed states (E* ∼ 0.6) (accounting for the photo-physics of the acceptor dye producing additional states, as described in Supplementary Figure S3); and one low FRET state in which the acceptor is bleached (E* ∼ 0.15). The dwells in the fingers-open and fingers-closed conformation were extracted from these HMM fits by classifying events according to: 0.3 < E* ≤ 0.53 as fingers-open; 0.53 < E* ≤ 0.8 as fingers-closed; and 0 < E* ≤ 0.3 as donor-only KF.

### Data filtering

Fluorescence intensity traces were filtered to minimize contributions from (short-lived) KF–surface adsorption events (rather than KF–DNA binding) by restricting data analysis to events with lifetimes of longer than 0.5 s, unless otherwise indicated.

For studies of the binary complex and mismatch complexes, further filtering was performed to remove residual KF-surface adsorbed complexes. This was performed by quantifying the conformation of KF molecules in the presence and absence of surface-immobilized DNAs, and using this quantification to remove non-DNA-bound KF molecules from subsequent analysis. Each KF complex was classified by the fraction of time spent in the fingers-closed conformation (calculated by removing bleached acceptor dye frames (E* < 0.35), and calculating the fraction of remaining frames with E* > 0.55; Supplementary Figure S4A). In the absence of DNA, transiently adsorbed KF–surface complexes demonstrated a broad, heterogeneous distribution of conformations (Supplementary Figure S4B). In the presence of DNA, this broad distribution consisted of only ∼10% of binding events. We assigned the remaining 90% of events to KF–DNA complexes, as they showed a distinct, predominantly fingers-open, behavior (Supplementary Figure S4A)

Filtering residual KF–surface immobilized events did not have a significant effect on our results, as can be seen by comparing histograms of dwells in the fingers-open conformation before (Supplementary Figure S4C) and after (Figure 3B) filtering. Comparable fingers opening and closing rates were extracted when this filtering was not performed. The main difference being that the fingers-open dwell histogram was fit to a double exponential, which represented the additional contribution from the KF–surface binding events (Supplementary Figure S4C). Ternary complex kinetic data were extracted without this filtering, with the rates extracted by fitting an additional exponential component representing the KF–surface bound kinetics when it produced a distinct contribution (i.e. at 0 and 0.1 μM dTTP; Supplementary Figure S5).

**Figure 2.**
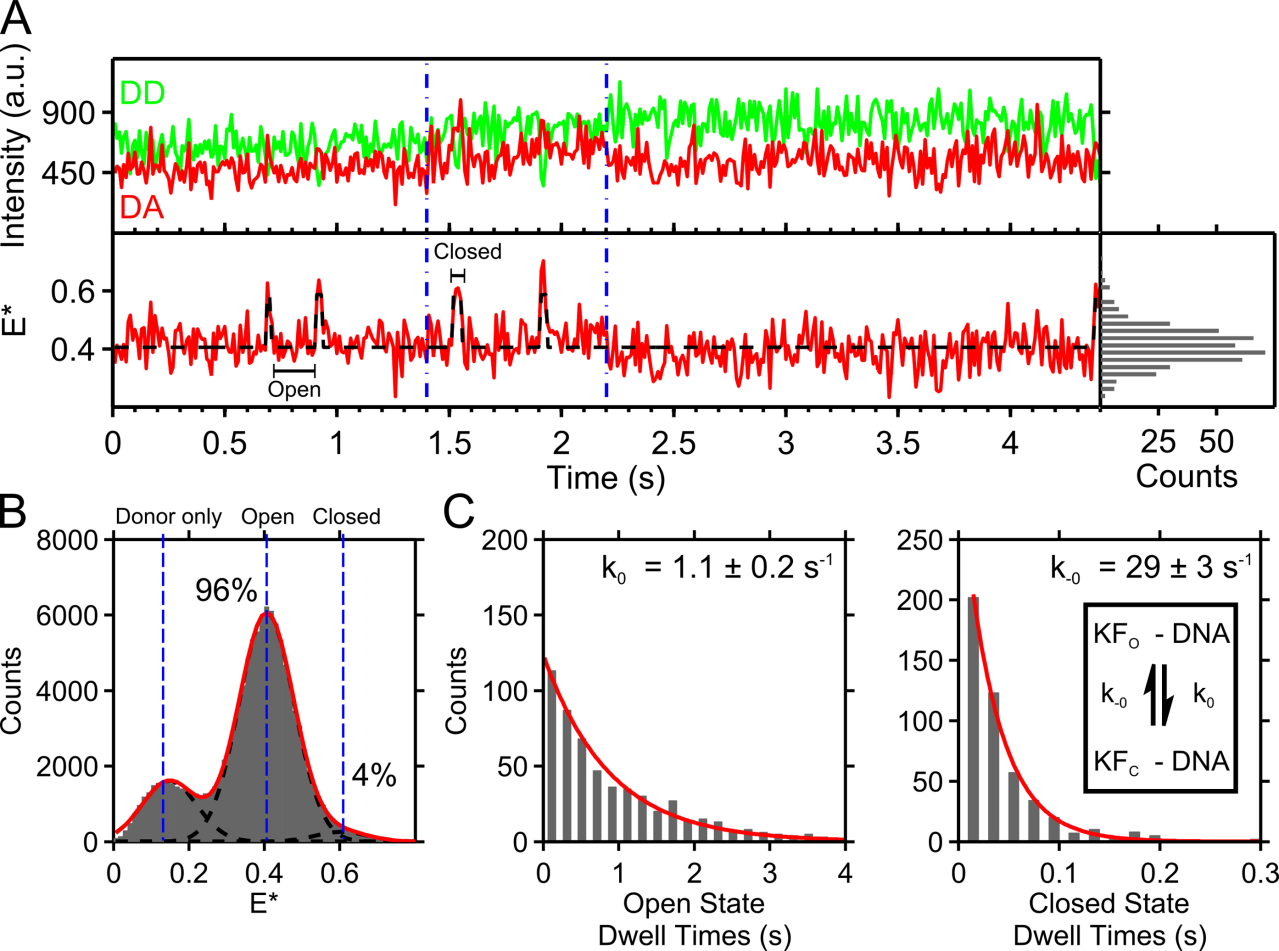
Fingers conformations of the binary complex. Data collected for 20 min under green-only excitation (1.5 mW) using 10-ms exposure times from KF binding to 96 separate DNA molecules. (**A**) Fluorescence intensity trace of three concatenated KF–DNA binary complexes, separated by blue lines. Lower panel shows FRET values alternating between E* ∼ 0.4 (fingers-open) and E* ∼ 0.6 (fingers-closed) with corresponding DD-DA fluorescence anti-correlation in the upper panel. Closing is slow relative to opening. A histogram of the E* values is seen on the right hand panel. The black dotted line is the hidden Markov model (HMM) fit. (**B**) Histogram of E* values from binding events across data-filtered KF–DNA complexes (see ‘Materials and Methods’ section). Black dotted lines are Gaussian fits to the closed state, open state, and donor-only population, with overall fit in red. Open and closed state occupancies are noted. (**C**) The dwell times in the fingers-open and fingers-closed states as extracted using HMM for data-filtered KF–DNA complexes. Single exponential fits quantify the slow closing rate (*k*_0_) and faster opening rate (*k*_-0_). The equilibrium due to the interconversion between the binary complex open (KF_O_–DNA) to closed (KF_C_–DNA) conformations is shown as an inset in the right panel.

**Figure 3.**
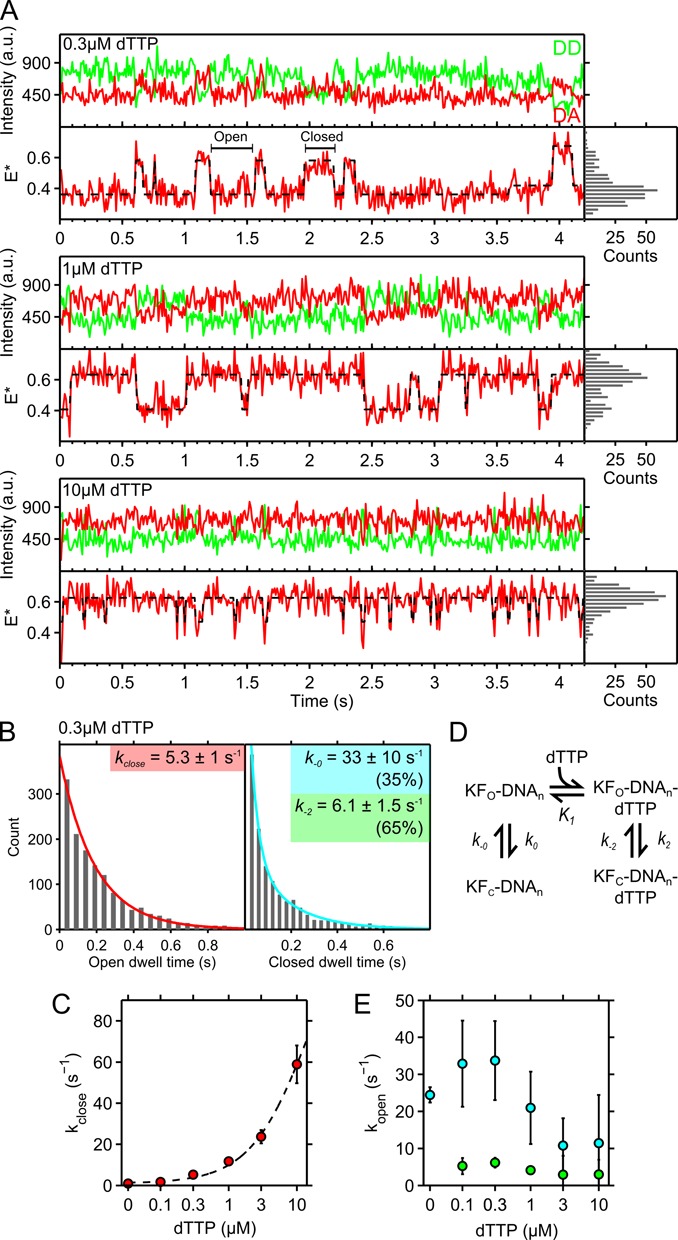
Fingers conformations in the presence of complementary nucleotide. Data was acquired for 20 min using continuous green excitation at 1.5 mW, with a 10-ms frame time from binding to 100, 78, 100, 89, 78 DNA molecules at 0.1, 0.3, 1, 3 and 10 μM dTTP concentrations, respectively. (**A**) Fluorescence intensity and E* traces of KF–DNA complexes in the presence of 0.3, 1 and 10 μM of complementary nucleotide (A-dTTP). E* histograms are in the right hand panels. Slow photophysical fluctuations (∼ 0.5 s^−1^) in ATTO647N lead to small shifts in E*, as seen in the final section of the 0.3 μM trace (see Supplementary Figure S3). (**B**) Fingers-open and fingers-closed dwell times in the presence of 0.3 μM dTTP. The open dwells fit to a single exponential (red) extracting an observed closing rate (*k*_close_; see Equation (1), main text). The closed dwell times fit to a double exponential, with a fast and a slow opening rate, representing binary (*k*_-0_) and ternary (*k*_-2_) complex opening. (**C**) The observed fingers-closing rates as a function of complementary nucleotide concentration (Supplementary Figure S5). Error bars represent the 99% confidence intervals on the fits. Data are fit to an equation (Equation 1, main text) derived from the model shown in panel D (gray dotted line). (**D**) Reaction schematic illustrating binary and ternary complex dynamics. The polymerase is shown in its open (KF_O_) or closed (KF_C_) states, bound to DNA, with or without complementary nucleotide (dTTP). (**E**) In the presence of nucleotides, the fingers-opening is described by two rates: the binary complex opening (green) and the ternary complex opening (blue) rates, extracted from dwell time histograms seen in Supplementary Figure S7. The extracted rates of the ternary complex opening remain similar until 3 μM, at which missed events contribute to an apparent lengthening of the dwells in the closed state.

## RESULTS

### An intra-protein FRET assay for studying fingers conformations in real time

To obtain extended, real-time observations of fingers conformational changes at the single-molecule level, we observed complexes of labeled KF bound to surface-immobilized DNA substrates. We monitored the FRET efficiency between a FRET donor at the tip of the KF fingers and a FRET acceptor at the base of the KF thumb (Figure 1A), thereby observing closing and opening of the fingers of single KF molecules. By using dideoxy-terminated primer DNA, we prevented DNA extension in the presence of nucleotides, thus focussing on conformational changes and equilibria preceding the chemical step (Figure 1B).

We first immobilized fluorescently labeled, primer-template DNA molecules on glass surfaces via biotin–neutravidin interactions (Figure 1C). The DNAs were localized via their fluorophores (which were subsequently photobleached) using TIRF imaging combined with alternating-laser excitation (ALEX) of donor and acceptor fluorophores (see ‘Materials and Methods’ section) (22–24). During donor illumination, we obtained donor fluorescence upon donor excitation (DD) and acceptor fluorescence upon donor excitation (DA); these photon counts reported both on KF binding to DNA, and (via FRET) on the KF fingers conformation whilst bound (Figure 1D). During acceptor illumination, we obtained the acceptor fluorescence upon acceptor excitation (AA), which reported on KF binding, and checked directly for any acceptor photophysics which may complicate data interpretation (Figure 1D).

Binding of a donor–acceptor labeled KF (KF^DA^) to a surface-immobilized DNA molecule led to a simultaneous fluorescence intensity increase in DD, DA and AA fluorescence counts (see event at ∼10.5 s in Figure 1D); the DD and DA counts were used to calculate the FRET efficiency reporting on the fingers conformational status. Dissociation of KF from the DNA led to simultaneous loss of all three photon counts (see event at ∼16 s in Figure 1D). Periods of increased fluorescence were assigned as ‘KF binding-events’, and their dwell-times were extracted (see ‘Materials and Methods’ section). Our KF protein sample also contained a population of singly labeled KF molecules, which we distinguished using ALEX; specifically, binding of donor-only or acceptor-only KF molecules (KF^D^ and KF^A^, respectively) resulted in an increase in either the DD or the AA channels alone, but no change in the DA channel (Figure 1D, ∼26 and ∼41 s, respectively). Doubly labeled polymerases accounted for ∼60% of binding events; singly labeled polymerases accounted for the remaining ∼40%. When we used ∼16 nM KF, we observed a similar behavior, with the additional feature of ∼5% of frames showing twice the expected fluorescence intensity, likely caused by either binding of a KF dimer to DNA (25), or nearby KF–surface binding (Supplementary Figure S2).

To analyze the dwell-time of KF on DNA, we looked at the distribution of the durations of the binding events. The distribution was bi-exponential (Figure 1E), with a major population (80%) of stable binding, and a minor population (20%) of unstable binding events with dissociation rates of *k*_off,long_ = 0.22 s^−1^ and *k*_off,short_ = 3.3 s^−1^, respectively. To identify the source of KF binding modes, we examined the KF non-specific surface adsorption without surface-immobilized DNAs. Although we observed much lower KF binding to the surface (Supplementary Figure S1A), we identified short binding events with a dissociation rate of *k*_off_ = 3.1 s^−1^ (Supplementary Figure S1B, left). We thus assigned the stable binding mode to KF–DNA specific binding, and the unstable binding to KF non-specific surface adsorption; we then confined our subsequent analysis to events lasting longer than 0.5 s to reduce the contribution of non-specific binding. Specific KF–DNA binding events were also clustered around the position of DNA substrates on the surface (as seen after localization analysis), a behavior not seen for the non-specific surface binding in the absence of DNA (Supplementary Figure S1C).

Within specific KF–DNA binding events, we observed the apparent FRET efficiency E* (‘Materials and Methods’ section) interconverting between states of E*∼ 0.4 and E*∼ 0.6 (Figure 1F), which we assigned to the fingers-open conformation (low-FRET) and fingers-closed conformation (high-FRET) respectively, in accordance with previous diffusion-based work (8,9). The use of ALEX also showed slow (∼0.3 s^−1^) fluorescence changes in the AA channel (likely due to acceptor photophysics), which led to small changes (ΔE*∼ 0.08) in the E* of the open and closed states (Supplementary Figure S3); we adjusted our state-finding algorithm to account for such effects (‘Materials and Methods’ section). Having established that our assay could detect real-time interconversions between the different conformational states of the fingers sub-domain, we characterized the kinetics of these dynamics in the absence of nucleotides, as well as in the presence of complementary or non-complementary nucleotides.

### Polymerase binary complex demonstrates intrinsic dynamics

We first examined the KF–DNA binary complex for conformational dynamics. The fingers subdomain in the binary complex was found primarily in the open state (Figure 2A, first and second binding event; open state at E* ∼ 0.4), and only transiently occupied the closed state (Figure 2A, first and second binding event; closed state at E* ∼ 0.6, as seen at ∼0.7 and ∼0.9 s). Some binding events do not show transitions to the closed state for several seconds (Figure 2A, third binding event). After data filtering to remove any residual KF surface-binding events (‘Materials and Methods’ section), we quantified binary-complex dynamics by fitting a HMM to FRET time traces (Figure 2A, black dotted line; also see ‘Materials and Methods’ section) and extracted the dwell times both in the fingers-open and fingers-closed conformations. The KF–DNA binding events were strongly biased toward the fingers-open conformation (96% fingers-open occupancy; see E* histogram, Figure 2B). The dwell-time histograms for the open and closed conformations were described well by single exponentials, consistent with a single rate-limiting step for closing and opening. Fingers-closing is slow (*k*_0_ = 1.1 ± 0.2 s^−1^), whereas fingers-opening is fast (*k*_-0_ ∼ 29 ± 3 s^−1^) (Figure 2C). The ratio of these rates defined an open-state occupancy of 96%, in exact agreement with our TIRF FRET-histogram-based results (Figure 2B). Our results on immobilized binary complexes also agree with the fingers-open occupancy measured using diffusion-based ALEX (81% open; Supplementary Figure S4F (9)). To verify that the binary dynamics were not highly dependent on the templating base, we repeated our experiments using a DNA sequence with a template C. We observed similar rates for fingers opening and closing with this C-template DNA as for binary complexes with an A template (*k*_0_ = 1.2 ± 0.6 s^−1^; *k*_-0_ ∼ 23 ± 3 s^−1^) (Supplementary Figure S6).

### Prechemistry fingers dynamics in the presence of complementary nucleotide

We then studied the fingers dynamics of the ternary complex (Figure 3A) by monitoring the FRET states of KF molecules bound to surface-immobilized DNA in the presence of increasing concentrations of complementary dNTP (in this case, dTTP). At low dTTP concentrations (e.g. 0.3 μM; Figure 3A, top panel), KF sampled mainly the low FRET state (E* ∼ 0.4); this state is likely to correspond to the fingers-open conformation of the binary complex since, for low dTTP concentrations, KF waits significant time for a complementary dNTP to bind and lead to fingers-closing. KF also sampled the high-FRET state (E* ∼ 0.6) that was, in most cases, much more stable (showing dwell times of >200 ms) than in the binary complex (compare Figure 2A with Figure 3A). As we increased the concentration of complementary dNTP, we observed two effects: the dwell times in the open state decreased, and the closed state occupancy increased (Figure 3A, middle and lower panels), thus shifting the open–closed conformational equilibrium toward the closed state.

We then extracted the kinetics of fingers-opening and closing for binding events longer than 0.5 s. The dwell-time histograms for the open conformation fit well to single exponentials (Figure 3B), except for our lowest dTTP concentration (0.1 μM), which fit better to a double-exponential decay (Supplementary Figure S5B, with the second rate arising from KF non-specific surface binding; see ‘Materials and Methods’ section and Supplementary Figure S4). As expected, the rates for fingers-closing (*k*_close ­_) increased with nucleotide concentration, ranging from 1.5 s^−1^ at 0.1 μM dTTP to ∼60 s^−1^ at 10 μM dTTP (Figure 3C). At concentrations higher than 10 μM dTTP, the closing rates were too fast to be extracted reliably.

To interpret the observed dNTP-concentration dependence of *k*_close_ (Figure 3C), we devised a simple kinetic model that accounted for binary complex dynamics, nucleotide binding, and subsequent ternary complex dynamics (Figure 3D). This model resembled one we described recently to infer the occupancy of open, closed and partially closed conformations of the KF fingers from smFRET data within diffusing molecules (9). Since we cannot reliably resolve the open and partially closed conformations (which differ only by E* ∼ 0.05, a FRET difference hard to resolve within a single smFRET time-trace using TIRF, see (19)), we regard our initial nucleotide-bound complex (KF_O_–DNA–dTTP in Figure 3D) as a composite of the open ternary complex and the partially closed ternary complex. Our new model includes both binary-complex dynamics (with rates of *k*_0_ and *k*_-0_ for closing and opening, respectively) and ternary-complex dynamics (with rates of *k*_2_ and *k*_-2_ for closing and opening, respectively) upon binding of a complementary nucleotide with an affinity characterized by the equilibrium association constant *K*_1_; the affinity of complementary nucleotide thus modulates the fingers interconversion rates. The experimentally accessible concentration-dependent rate of fingers closing, *k*_obs, close_, was fit using the equation (see *SI*):

(1) \begin{equation*} k_{\rm obs,close}=(k_{0}+{\rm [dTTP]}k_{2}K_{1}) \left/ (1+{\rm [dTTP]K_{1}}) \right. \end{equation*}

Fixing *k*_0_ to the value obtained for the binary complex (1.1 s^−1^), we extracted a *K*_1_ of 0.06 **±** 0.04 μM^−1^ (corresponding to a *K*_d_ of ∼16 μM) for the complementary dNTP (A-dTTP), and a fingers-closing rate *k*_2_ of 150 **±** 70 s^−1^. Our measurement of *k*_2_ was in excellent agreement with previous measurements of fingers-closing rate (140 s^−1^; see (26)).

To extract the rate of fingers-opening in the KF–DNA complexes in the presence of increasing dNTP concentrations, we fit the dwell-time histograms of the closed conformation using two exponential-decay components: one with a fast opening rate *k*_-0_ assigned to binary-complex fingers opening, and a second, slower opening rate *k*_-2_ assigned to ternary-complex fingers opening (see Figure 3B, right panel, for the 0.3 μM dTTP data). This analysis was reliable at dNTP concentrations below 3 μM; at higher concentrations, fast fingers-closing led to missed dwells in the open state, artificially lenthening the closed-state dwells (Figure 3A, bottom panel). The fast opening rate (*k*_-0_ = 33 ± 10 s^−1^, blue circles, Figure 3E) was in excellent agreement with the rate extracted for the pure binary complex (29 s^−1^; Figure 2C). In contrast, the opening rate from the ternary complex (*k*_-2_ = 6.1 ± 1.5 s^−**1**^, green circles, Figure 3E) was much slower than the opening rate of the binary complex, presumably due to stabilization of the closed state by the presence of the complementary nucleotide.

To verify that the general observation of rapid fingers-closing and slow fingers-opening in the presence of complementary nucleotide was not affected by the base-pair used, we repeated our experiments using a DNA sequence with a template C. We observed that fingers-closing with a dGTP-C pair was ∼2.5-fold faster at 0.3 μM and 1 μM dGTP than for the A-dTTP pair, and closing events were too fast to be resolved using HMM at 10 μM dGTP (Supplementary Figure S8). These results are consistent with the higher ground-state binding affinities for C-dGTP pairs observed in previous work (9). Fingers opening rates were similar to those measured using an A-dTTP pair.

### Fingers occupy an intermediate FRET state in the presence of non-complementary dNTP

**Figure 4.**
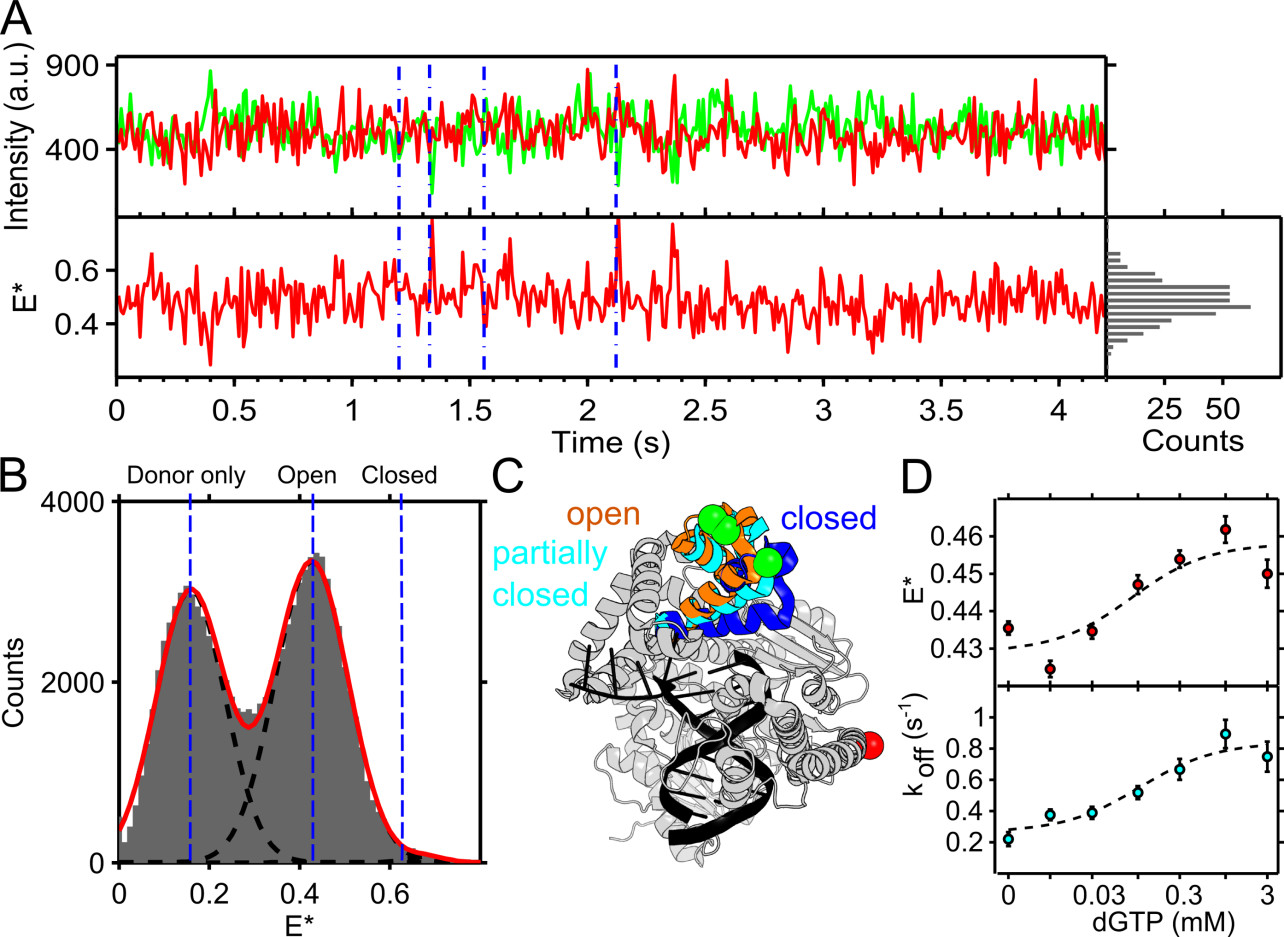
Fingers conformations in the presence of non-complementary nucleotides. Data collected under green-only excitation at 2 mW intensity and 10-ms frame time for panels A and B, and 0.3 mW intensity and 100-ms frame time for panel D, from KF binding to 144, 140, 143, 164, 153, 117, 141, DNA molecules at concentrations of 0, 0.01, 0.03, 0.1, 0.3, 1, 3 mM dGTP respectively. (**A**) Fluorescence intensity trace of five concatenated KF-DNA complexes in the presence 100 μM of dGTP, separated by blue lines. Lower panel shows FRET rare interconversions between E* ∼ 0.4 (fingers-open) and E* ∼ 0.6 (fingers-closed). A histogram of the E* values is seen on the right hand panel. (**B**) Histogram of E* values at each frame across data-filtered KF–DNA complexes (see ‘Materials and Methods’ section). Black dotted lines are Gaussian fits to the closed state, open state, and donor only population, with overall fit in red. (**C**) *Bst* polymerase structures illustrating the position of the fingers in the open (PDB structure 1L3U), partially closed (3HPO) and closed states (1LV5), respectively. The dye positions are indicated as in Figure 1A. (**D**) Upper panel: low-FRET state peak position against dGTP concentration. Lower panel: KF–DNA slow dissociation rate (*k*_off)_ as extracted from dwell time histograms (Supplementary Figure S9) against dGTP concentration. Dotted lines were added as visual guides.

We also observed the fingers conformational profile in ternary complexes with a non-complementary dNTP; specifically, we examined the A-dGTP mispair, which we have previously characterized in solution (8,9). Intensity traces demonstrated that the closed state was rarely populated in the presence of non-complementary nucleotide (Figure 4A), consistent with our previous work; the FRET histogram confirmed this finding (Figure 4B; binding events filtered as for the binary complex). In addition, the presence of the A-dGTP mispair decreased the stability of the KF–DNA complex (Figure 4A), with the KF–DNA dissociation rates increasing from 0.22 **±** 0.05 s^−1^ without dGTP, to 0.75 **±** 0.1 s^−1^ in the presence of 3 mM dGTP (Figure 4D; for dwell-times histograms of the KF–DNA complex, see Supplementary Figure S9A). The rare interconversions and shorter dwell times of KF on DNA complicated the extraction of kinetic information using HMM. Increased KF-DNA dissociation rate in the presence of non-complementary nucleotide has been seen previously, and may reflect an unstable structural state (27).

In prior work, we identified a novel partially closed KF conformation populated in the presence of non-complementary nucleotide. This conformation had a FRET value intermediate between the open and closed states, and was assigned to partially-closed conformation of the KF fingers seen in crystal structures (Figure 4C) (8,28). Consistent with our previous results, we observed a FRET increase from E* ∼ 0.43 in the fingers-open state, to a state of E* ∼ 0.46 at mM concentrations of non-complementary nucleotide (dGTP; Figure 4B and Supplementary Figure S9B). However, we did not observe interconversions between in the fingers-open and fingers-partially-closed conformations in individual FRET time-traces of KF (Figure 4A). This is not surprising since, based on our solution-based work, the FRET difference between the open and partially closed state is small (only 0.04, due to an increase from 0.40 to 0.44), and the interconversion kinetics may be rapid (due to the structural similarity of the states) compared to our temporal resolution.

## DISCUSSION

### Direct observation of polymerase conformational changes

Our previous single-molecule FRET studies on diffusing polymerase complexes highlighted the importance of conformational changes of the fingers subdomain for polymerase fidelity, but did not access directly the kinetics of such changes. Since the observation time for each molecule in diffusion-based experiments was limited to a few milliseconds, interconversion rates could only be inferred under a set of assumptions (8–11). Here, we observed the conformational changes of the fingers subdomain for single binary and ternary complexes and obtained the rates for fingers-opening and closing transitions.

Our results confirmed the presence of conformational dynamics in the binary complex, and showed that the fingers-closing rate is relatively slow (*k*_0_ = 1.1 ± 0.2 s^−1^), while fingers-opening is rapid (*k*_-0_ ∼ 29 ± 3 s^−1^). Our values improve significantly on previous estimates of *k*_0_ ∼ 15 s^−1^ and *k*_-0_ ∼ 85 s^−1^, derived from fitting FRET distribution of brief KF snapshots to a model based on two interconverting conformations (9). We note that no such fluctuations were seen in T7 DNA polymerase complexes (12), either due a different equilibrium, or due to the lower temporal resolution (200 ms versus 10 ms in our work) and the less direct nature of the previous assay, which was based on an environmentally sensitive dye.

### Balance between fingers-opening, fingers-closing and reaction rate-limiting step

We also observed that the conformational equilibrium of ternary complexes shifts toward the closed conformation with increasing complementary nucleotide concentration. Using a model based on the properties of binary and ternary complexes, we obtained a fast closing rate of *k*_2_ = 150 s^−1^ for KF in ternary complexes in the presence of complementary nucleotide, in excellent agreement with the rate observed for KF using bulk stopped-flow FRET measurements (140 s^−1^; (26)). In contrast, the opening rate for the ternary complex (*k*_-2_ = 6 s^−1^) is ∼25-fold slower, heavily biasing the equilibrium toward the closed state upon binding of the complementary dNTP. We considered these rates in the context of a simplified reaction scheme, including a conformational change and chemistry, but no binary complex dynamics:

(2) \begin{equation*} \begin{array}{*{20}c} {{\rm KF}_{\rm O} \cdot {\rm DNA}_{n} \mathop \rightleftharpoons \limits_{k_{ - 1} }^{[{\rm dTTP}]k_1 } {\rm KF}_{\rm O} \cdot {\rm DNA}_{n} \cdot {\rm dTTP}} \\ {\mathop \rightleftharpoons \limits_{k_{ - 2} }^{k_2 } {\rm KF}_{\rm C} \cdot {\rm DNA}_{n} \cdot {\rm dTTP}\mathop \to \limits^{k_3 } {\rm KF} \cdot {\rm DNA}_{{n} + {\rm 1}} + {\rm PP}_{\rm i} } \\ \end{array} \end{equation*}

These two rates ensure that after nucleotide binding and fingers-closing, nucleotide incorporation is overwhelmingly likely to occur, since the rate of fingers-opening (a pre-requisite for nucleotide release *before* chemistry) is much slower (6 s^−1^) than the rate-limiting step in the forward direction (with rate constant *k*_3_ ∼ 47 s^−1^, based on single-turnover nucleotide incorporation rates by KF for different dNTPs (26,29–30)). This large rate difference makes fidelity less dependent on the exact rate constant of the rate-limiting step. This fidelity feature was initially shown for T7 DNA polymerase (13) (see also *SI* for derivations), which also exhibits a rapid conformational change upon binding of a complementary nucleotide; the slow reversal of this change delays nucleotide release, biasing the reaction toward the productive forward direction. Although forward biasing is less pronounced in KF than for T7 DNA polymerase, the limit for the catalytic efficiency for complementary nucleotide calculated for T7 DNA polymerase, in which the rate-limiting step becomes less important (*k*_cat_/*K*_m_ = *K*_1_*k*_2_), also applies to KF within a factor of (1+ *k*_-2_/*k*_3_) = 0.9 (see *SI*). Our results suggest that the conformational change identified in T7 DNA polymerase corresponds to the fingers-closing transition; the similarities between KF and T7 DNA polymerase suggest that this kinetic balance may be a general mechanism for polymerases.

### Induced fit versus conformational selection

Our results provide a clear answer on whether DNA polymerase I uses an induced-fit or a conformational selection mechanism to recognize and discriminate nucleotides. Such mechanistic models assign different roles and importance to the presence of protein conformational dynamics and states present in the absence of the ligands, and have been the recent focus of a lively debate (31–33).

According to the induced-fit model, complementary nucleotides bind to the fingers-open conformation and induce full fingers-closing; despite this being the leading model for substrate recognition, it has not been explicitly demonstrated. An alternative model supports recognition via conformational selection, wherein the KF–DNA binary complex dynamically interconverts between open and closed conformations, with the incoming complementary nucleotide binding and stabilizing the closed conformation. Our results distinguish conclusively between the two models on the basis of the dNTP-concentration-dependence of the closing rate of the ternary complexes: the induced-fit model predicts an increase in the fingers-closing rate with increased nucleotide concentration, whereas conformational selection predicts that the fingers-closing rate will remain constant and equal to the closing rate (*k*_0_) of the binary complex (31). As we observed a pronounced increase of the rate upon increasing the concentration of (correct) dNTPs (Figure 3D), our data strongly supports the induced-fit model.

### Partial-closed conformations and polymerase fidelity

Our results also offer new insights in polymerase fidelity through the analysis of KF conformations in ternary complexes with non-complementary dNTPs. In experiments using a A-dGTP mispair on ternary complexes, we observed KF fingers being predominantly open (or partially closed, depending on nucleotide concentration), meaning that fingers opening is much faster than fingers closing.

Previous work has identified a partially-closed conformation intermediate between the fingers-open and closed conformations (8–9,28). Using TIRF microscopy, we were not able to resolve this state in real-time, suggesting that the structural inter-conversions between the states are faster than our corresponding time resolution (10 ms), or that our FRET pair is not sensitive to the small distance change when used at the level of a *single* immobilized molecule. In contrast, similar work by Berezhna et al. resolved an intermediate FRET state (which was assigned to the partially closed state) in single-molecule FRET traces of KF at a much lower time resolution (100 ms; (15)). The FRET pair used by Berezhna *et al*. has a distance sensitivity similar to our FRET pair (Alexa-Fluor 488/594 Förster radius of *R*_o_ ∼ 60 Å (15)) compared to the *R*_o_ ∼62 Å for Cy3b-ATTO647N (34)), suggesting that this state should be resolvable in our assay too, provided it is associated with a change in fingers conformation. The discrepancy between our work and the work of Berezhna *et al*. may be due to the labeling positions of the FRET pairs used. Whereas our intra-protein FRET ruler directly measures fingers conformations, the assay used by Berezhna *et al*. infers the fingers conformation by monitoring the distance between a fluorophore on the DNA and a fluorophore attached to the protein. Thereby, the latter FRET signals may be affected by transient intermediates and alternate binding orientations that have been observed when using protein–DNA FRET pairs (16,35–36). Further complications in the Berezhna work may arise from the use of a DNA substrate with both 3′ and 5′ single-stranded overhangs, providing multiple potential binding sites for KF.

To get an estimate of KF fidelity from our current and previously measured rates and equilibrium constants, we can use the discrimination ratio *D*, defined as the ratio of catalytic efficiencies (*k*_cat_/*K*_m_) for complementary and mismatching nucleotide (13,37–38). As for T7 DNA polymerase (13), the discrimination for KF reduces to$D=K_{1}k_{1}{\left/ K_{1}^{'}K_{2}^{'}k_{3}^{'} \right.}$in our case (see *SI*). We then quantified KF fidelity in terms of the reaction sub-steps seen in Equation (2); these steps include: nucleotide binding before fingers closing; fingers-closing; and chemistry. Binding of the complementary (A-dTTP) and the non-complementary nucleotide (A-dGTP) in the open state, before closing, contributes a factor of ∼10 to fidelity (based on *K*_1_/*K′*_1_ with *K*_1_ ∼0.2 μM^−1^ and *K′*_1_ ∼0.02 μM^−1^ values from our work and previous work (9)). This could possibly be related to the partially-closed state sampling the incoming nucleotide for complementarity with the templating base (28). Secondly, the fingers-closing rate directly contributes to fidelity by a factor of ∼140 (*k*_2_ = 140 s^−1^ (26)), perhaps a surprisingly large contribution. Inefficient closing around mismatching nucleotide meanwhile contributes a factor of ∼10 (*K′*_2_ = 0.09 for A-dGTP (9)). Ignoring other steps, the kinetics and thermodynamics of fingers-closing alone therefore contribute a factor of ∼1400 toward fidelity, a dominant contribution toward fidelity compared to initial nucleotide binding. Combining all steps together results in a total selectivity factor of 4200–14 000; this selectivity is lower than previously measurements of selectivity of A-dTTP over A-dCTP incorporation of 25 000–75 000 (39). This difference likely reflects the value of the chemical step rate (*k′*_3_) for the mismatching nucleotide which we could not include in our analysis because of missing literature values.

Finally, our new assay should allow real-time studies of DNA synthesis on extensible substrates, and can be extended to polymerases with mutator phenotypes and complexes with incorrect nucleotides.

## SUPPLEMENTARY DATA

Supplementary Data are available at NAR Online.

SUPPLEMENTARY DATA
