# Supplementary material for: Real-time single-molecule studies of the motions of DNA polymerase fingers illuminate DNA synthesis mechanisms
Source: Nucleic Acids Res. 2015 May 26;43(12):5998–6008. doi: 10.1093/nar/gkv547 (PMC4499156; doi:10.1093/nar/gkv547)
Supplement: SUPPLEMENTARY DATA [file supp_43_12_5998__index.html]

Real-time single-molecule studies of the motions of DNA polymerase fingers illuminate DNA synthesis mechanisms — SUPPLEMENTARY DATA 

# Real-time single-molecule studies of the motions of DNA polymerase fingers illuminate DNA synthesis mechanisms

## SUPPLEMENTARY DATA

- SUPPLEMENTARY DATA
