# Supplementary material for: Real-time single-molecule studies of the motions of DNA polymerase fingers illuminate DNA synthesis mechanisms
Source: Nucleic Acids Res. 2015 May 26;43(12):5998–6008. doi: 10.1093/nar/gkv547 (PMC4499156; doi:10.1093/nar/gkv547)
Supplement: SUPPLEMENTARY DATA [file supp_gkv547_nar-00094-m-2015-File006.pdf]

SUPPLEMENTARY FIGURES

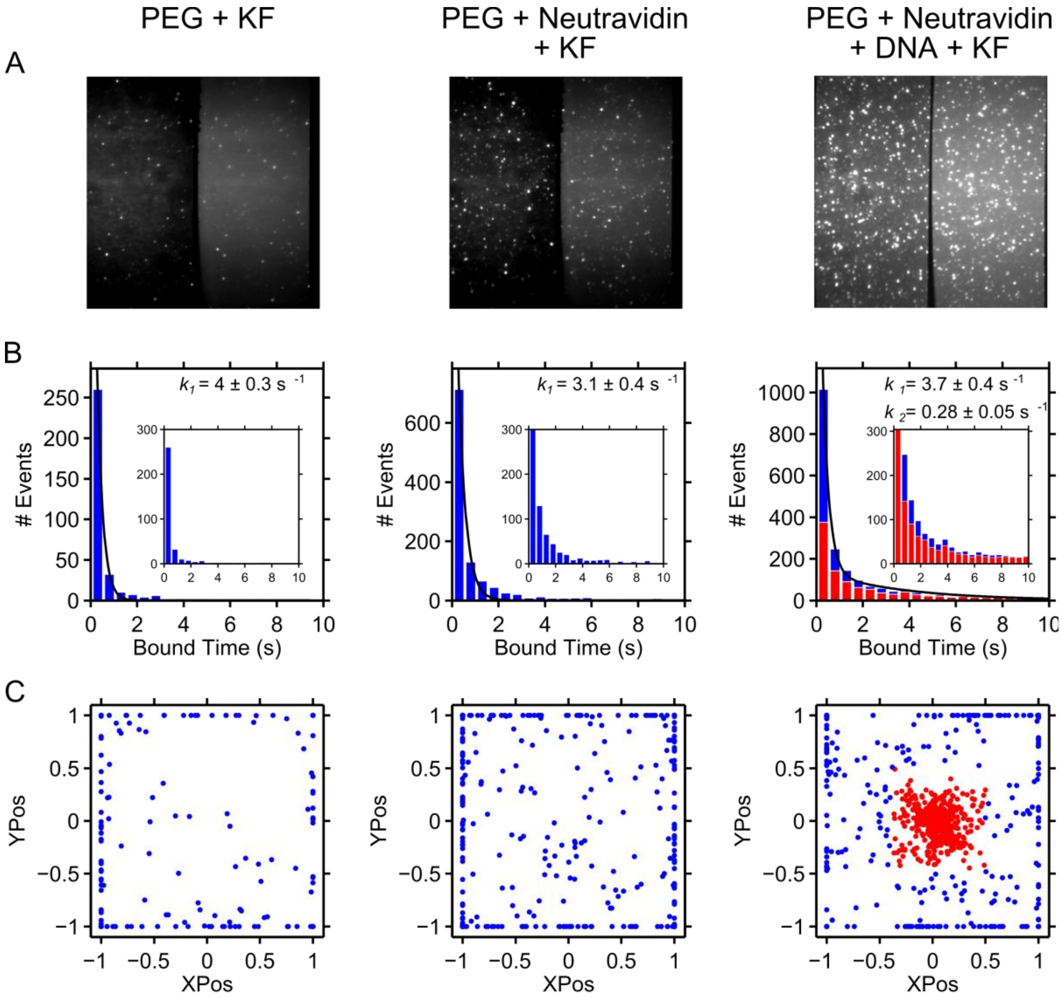

SI Figure 1. Binding of KF to surface-immobilized DNA relative to non-specific KF-surface binding. Transient immobilization of KF is achieved via binding to DNA which is surface-immobilized to PEG-passivated glass surfaces via a biotin-neutravidin-biotin interaction. We investigated the specificity of KF-DNA binding against KF-surface binding, by observing KF binding under various surface conditions: KF binding to PEG-passivated glass surfaces (left); KF binding to PEG-passivated glass surfaces treated with neutravidin (middle); and binding to PEG-passivated glass surfaces treated with neutravidin and with immobilized DNA (right).

A) Regions of a glass slide imaged over 80s demonstrate the immobilization of KF to these surfaces. Low KF binding is seen to PEG surfaces (left), with higher binding in the presence of neutravidin (middle), and much higher in the presence of DNA (right).

B) Histograms of KF dwells at randomly picked locations on PEG-passivated (left) and on neutravidin-treated slides (middle). KF dwells at localized DNAs are seen in the right panel. Dwells are collated from three field of views each. Insets show expanded y-axis. Unstable KF complexes (dissociation rate k_1) are seen to be caused by non-specific KF-surface binding exacerbated by neutravidin, whilst the longer binding events (dissociation rate k_2) only occur in the presence of DNA. Blue and red correspond with the localized positions specified below, loosely corresponding to KF-surface binding and KF-DNA binding respectively, according to the clustering around localized DNA positions.

C) Localized positions of the transient KF dwells relative to the randomly localized positions on PEG passivated (left) and neutravidin treated slides (middle) (units are in CCD pixels). Additionally, positions of transient KF dwells relative to localized DNA positions are seen (right). Clustering is seen around the centre in the presence of DNA, with red points assigned as KF-DNA binding (right), which are colour coded and linked to the dwell time histograms in b, demonstrating the KF-DNA binding events have distinctly longer dwell times than KF-surface binding events. Studies were performed using continuous wave (CW) excitation with a green laser power of 0.5 mW with a 40-ms frame time and 40 nM KF. Section c sampled from one field of view.

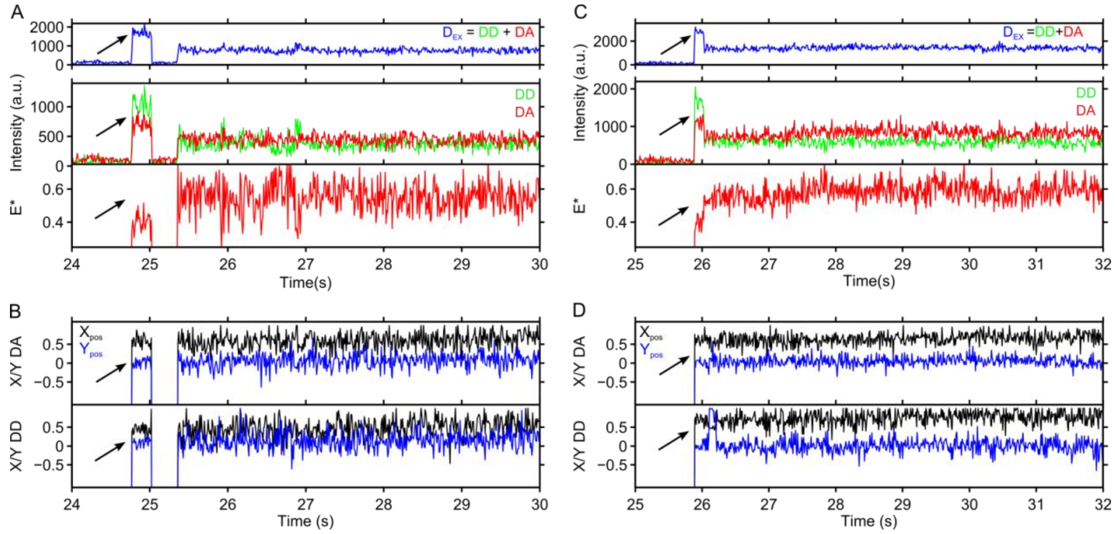

SI Figure 2. Single-molecule techniques allow the observation of rare events. Variations in fluorescence intensities of KF-DNA complexes suggest possible binding of two KF molecules simultaneously. Data was collected using continuous wave (CW) excitation with a green laser power of 0.3 mW with an 100-ms frame time, with a KF concentration of 4 nM and 1 µM dTTP.

A) At the first KF binding event (~25 s), the total intensity upon donor-excitation (D_{ex} = DD + DA) is about twice as high as the intensity of the second binding event (from ~25.3s). The FRET efficiency differs between the first and the second binding event (E* - red). The interpretation of the FRET efficiency in the first binding event, however, is difficult due to the higher number of fluorophores in close proximity and the unknown orientation of the molecules relative to each other. During the second binding event, KF is predominantly closed as expected.

B) To control for nearby KF-surface binding, which could cause such a doubling in intensity if the binding to the DNA and binding non-specifically to the surface happens simultaneously (an unlikely event), the X/Y positions of the localized PSFs (black/blue) relative to the initially localized DNA are displayed. The lack of change in X/Y position of KF between the high and low fluorescence intensity regions suggests that the second KF molecule is not binding to the surface, but rather to DNA. However, the possibility of KF surface-binding close (< 50 nm) to the DNA is not eliminated. Y position is centered around the localized DNA position (zero), but X position is offset by 0.5 pixels, caused by drift in the microscope after the initial DNA localization.

C) For a second position, we see similar behaviour to A,B as changes in total fluorescence intensity. Here, the initial fluorescence intensity D_{ex} begins a high level and drops to half the value, possibly triggered by the dissociation of one KF molecule from the homo-dimer bound to DNA or by bleaching of one of the two donors.

D) X/Y position of the PSF of KF does not change with the intensity changes. We note, however, a short shift in the DD position, but this occurs after the region of high intensity.

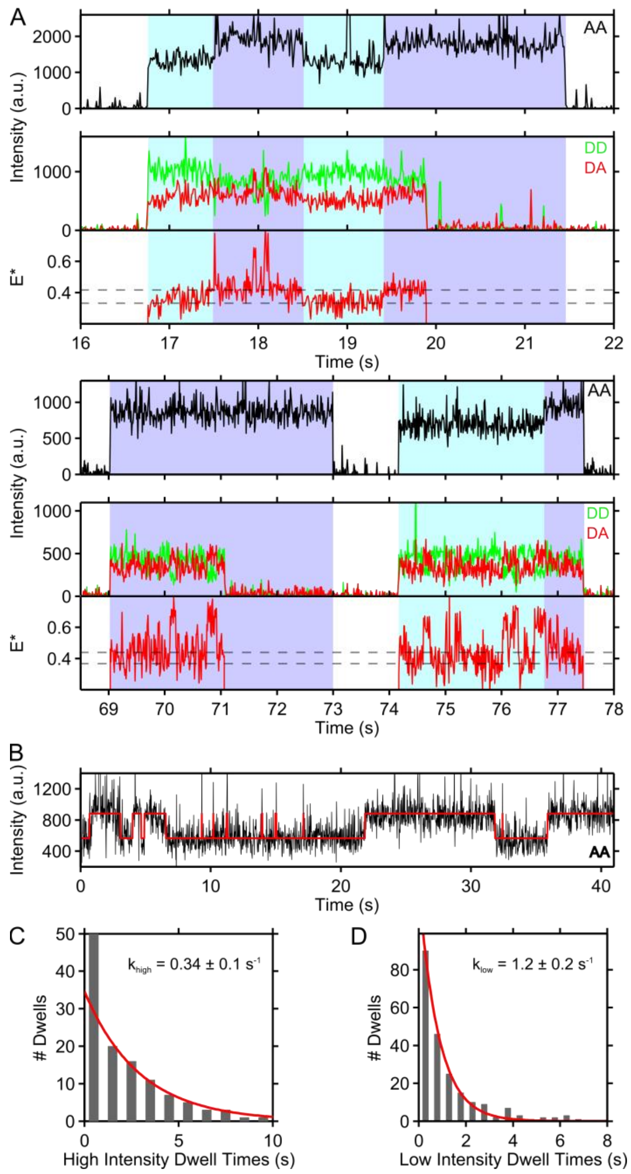

SI Figure 3. The effect of Atto647N photophysics on apparent FRET efficiencies.

A) Acceptor fluorescence intensity traces (AA – black) demonstrate two intensity values for Atto647N (blue and purple boxes), which are seen to correspond with small shifts in E* (dotted blue lines). Being smaller than the shift between open and closed states (ΔE*~0.08), this does not affect our ability to resolve these two states. Binding events at two DNA molecules are seen (upper and lower panels).

B) HMM fit to concatenated acceptor intensity traces at a single DNA molecule. Low and high intensity states are extracted, with the very short transitions to the higher state assigned to background noise.

C) HMM fits to KF-DNA complexes at 22 DNA molecules were used to extract the dwells in the high and low intensity states (intensity ratio 1:1.4).All binding events were concatenated for each DNA molecule. HMM analysis was conducted on traces which lasted longer than 7 seconds. The histogram of high intensity state dwells were fit to a single exponential extracting a high-to-low rate of k_{high}=0.34 s^{-1}. The first bin is ignored, as it was assigned to short noise events seen in b. Given the concatenation of the binding events used when applying the HMM, the rates extracted represent upper limits of interconversion rates.

D) Histogram of low intensity state dwells were fit to a single exponential, extracting an low-to-high rate of k_{low}=1.2 s^{-1}. This provides an upper limit on the low-to-high rate, which are additionally segmented by the short-noise events seen in C.

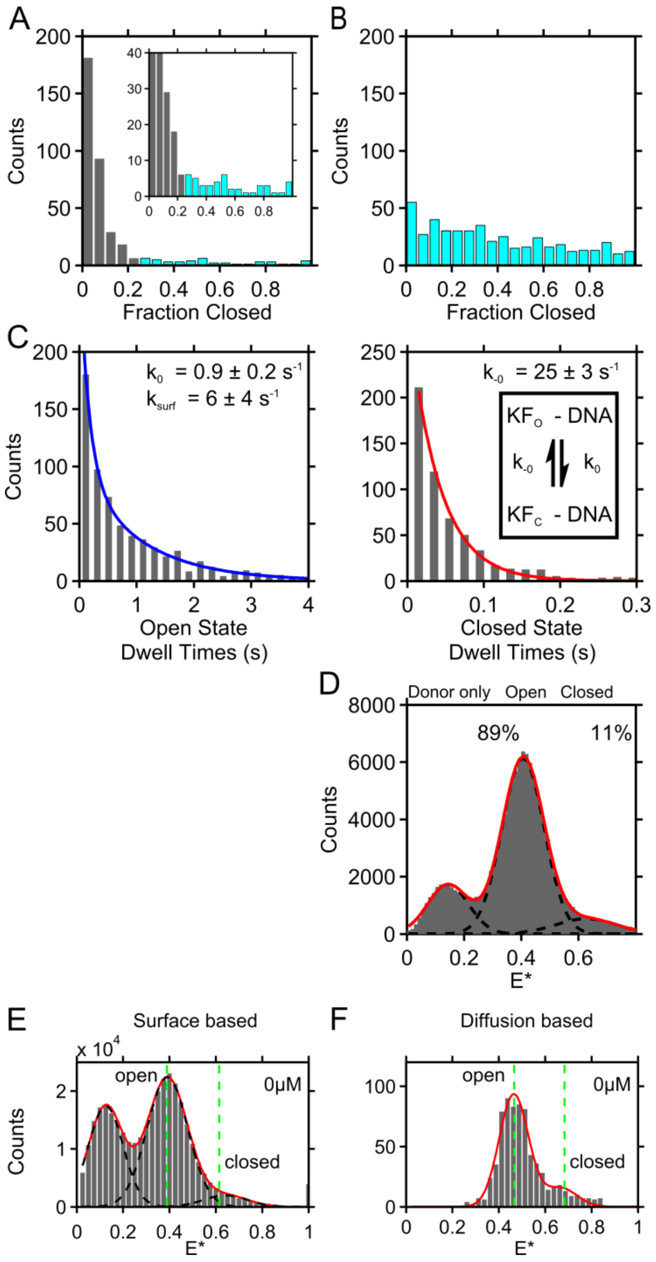

SI Figure 4. Characterising the contribution of KF-surface binding events to binary complex rate extraction. Residual KF surface complexes were removed by comparing the FRET behaviour of KF molecules non-specifically bound to the passivated surface in the absence of immobilized DNA, with the FRET behaviour of KF molecules bound in the presence of immobilized DNA.

(A, B) The fraction of time spent in the fingers closed conformation for each KF complex was calculated in the presence (A; inset shows zoom) and absence (B) of surface immobilized DNA. In particular, we first removed all frames corresponding to a donor-only signal after bleaching of the acceptor (using a threshold of $E^* < 0.35$), and calculated the fraction of frames in the closed state (using a threshold of $E^* > 0.55$) for all remaining frames. The majority of binding events exist in the open conformation (grey bins of Fraction Closed<0.25), with a small population of events (cyan) corresponding to the distribution seen in the absence of DNA.

C) Binary complex fingers-closing rates extracted without removal residual KF-surface complexes. The results are similar to those in Fig. 2, but the first peak is larger on the fingers-open dwell-time histograms, requiring a bi-exponential fit. The extracted rates reproduce the KF-DNA complex closing rate $k_0 = 0.9 \pm 0.2\ s^{-1}$, with the additional rate ascribed to KF-surface complexes closing $k_{surf} = 6 \pm 4\ s^{-1}$. (Left panel) Fingers-closed dwell-time histograms extract a fingers-opening rate of $k_{-0} = 25 \pm 3\ s^{-1}$ with a single exponential fit (right). The reaction scheme is shown in the right-hand panel.

D) A histogram of E* values at each frame, for all binding events, demonstrates that the KF-surface events do not dramatically change the state occupancy, compared to Fig 2B.

E) Histogram of E* values at KF-DNA binding events measured on a TIRF microscope, performed under CW excitation with a green laser power of 1.5mW with an 10 ms frame time, with a KF concentration of 4 nM. CW excitation results in an extra population at E* = 0.15 from donor-only molecules. Occupancies for the donor-only population, fingers-open population and fingers-close population were extracted by fitting 3 Gaussian profiles to the histograms, the position of the fits were constrained to E_1^* = 0.08 - 0.2, E_2^* = 0.39 - 0.45, E_3^* = 0.55 - 0.65.

F) Histogram of E* values of fluorescence bursts emitted as KF-DNA complexes diffuse through a focused laser beam. Measurements were performed as described (9) using an unlabelled version of the DNA construct in Figure 1B. We used the same Tris-buffer was in the TIRF experiments, but without the glucose oxidase and catalase oxygen scavenger system. Fingers-open and fingers-closed populations were extracted by fitting two unconstrained Gaussian profiles.

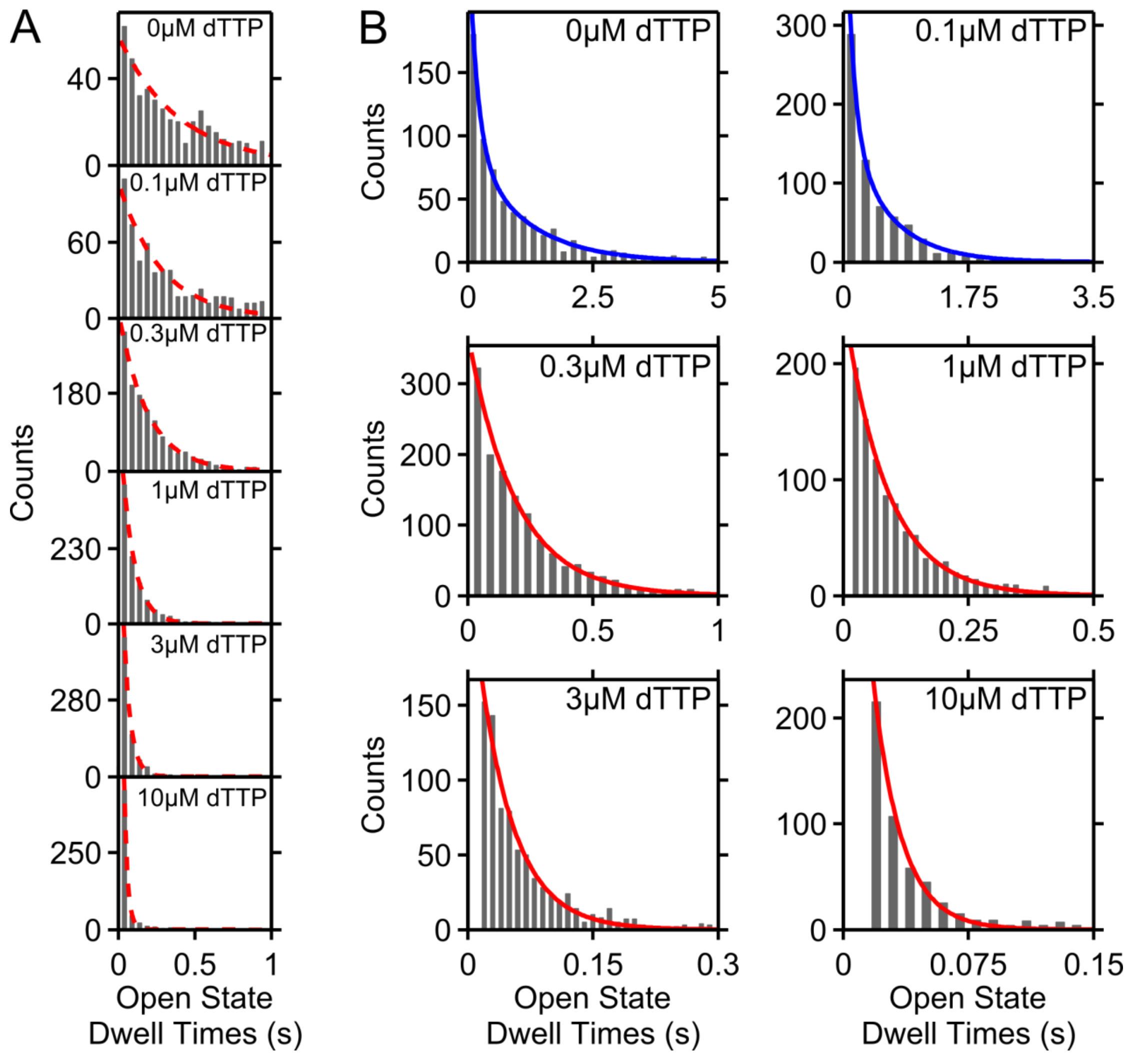

SI Figure 5. Fingers-open dwell times define the fingers-closing rates at different complementary nucleotide concentrations for KF.

A) Histogram of fingers-open dwell times demonstrating the decrease of dwell-times upon increasing nucleotide concentration (x-axis scaling held constant).

B) Same data as in panel A, but showing more clearly the fits of fingers-open dwell-time histograms, which are biexponential for dTTP = 0 and 0.1 μM, and single-exponential for other concentrations. Extracted rates are plotted in Fig 3D.

A

bio-5'-CCTCATTCTTCGTCCCATTACCATACATCC$_H$
3'-GGAGTAAGAAGCAGGGTAATGGTATGTAGGCTAGTA

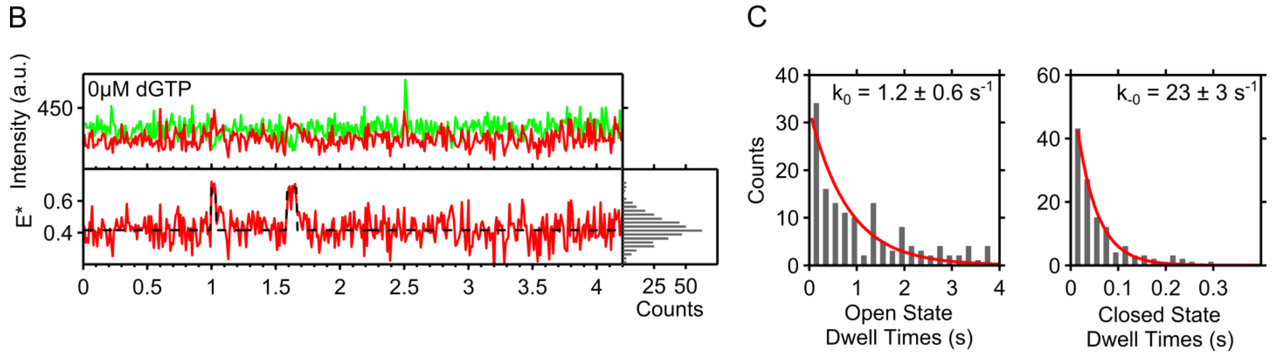

SI Figure 6. Fingers conformations of the binary complex with a template C.
Data collected for 5 min under green-only excitation (1.5 mW) using 10-ms exposure times.

A) Base-paired primer-template oligonucleotide used for these experiments and SI Fig 8. In the primer DNA, position -22 is labelled with a red fluorophore, the 5' terminal is biotinylated for surface immobilisation, and the 3' terminal base is dideoxy-modified (H) to prevent primer extension. The single-stranded overhang of the templating DNA is shown in blue. The first template base is a C.

B) Fluorescence intensity trace of a KF-DNA binary complex. Lower panel shows FRET values alternating between E*~0.4 (fingers-open) and E*~0.6 (fingers-closed) with corresponding DD-DA fluorescence anti-correlation in the upper panel. Closing is slow relative to opening. A histogram of the E* values is seen on the right hand panel. The black dotted line is the Hidden Markov Model (HMM) fit.

C) The dwell times in the fingers-open and fingers-closed states as extracted using HMM for KF-DNA complexes. Single exponential fits quantify the slow closing rate (k_0) and faster opening rate (k_{-0}).

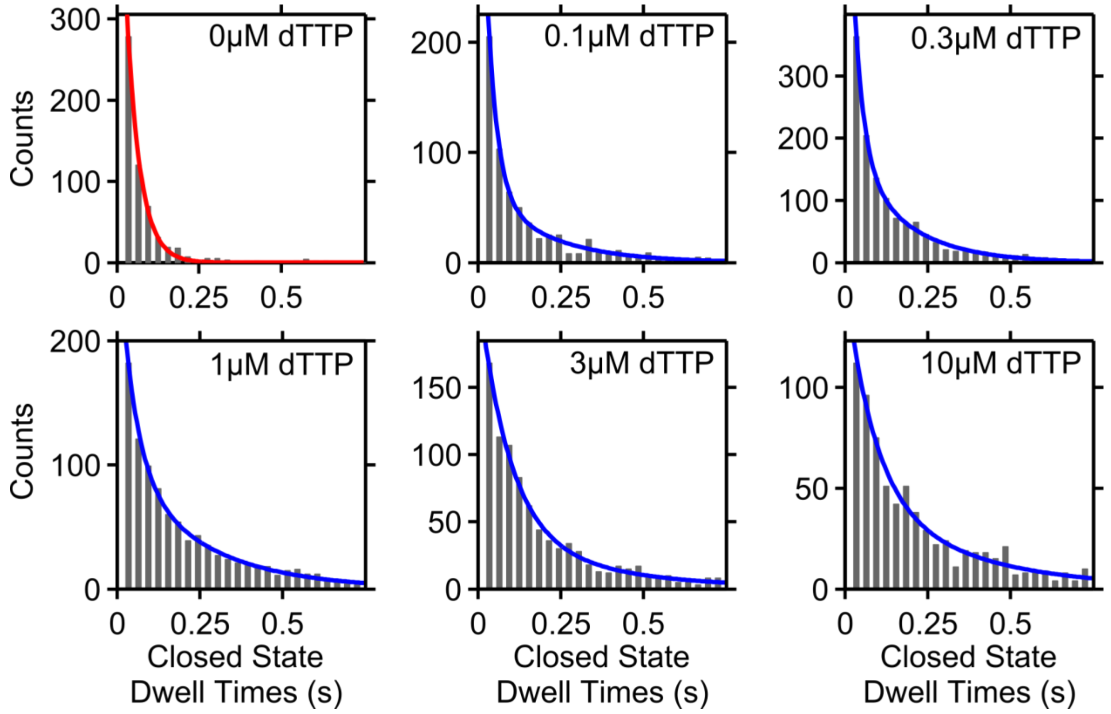

SI Figure 7. Fingers-closed dwells define the fingers-opening rates at different complementary nucleotide concentrations. Single exponential fits are seen in red, demonstrating the binary complex dynamics at 0 μM dTTP. Biexponential fits are in blue, representing the contributions of the binary (k_{-0}) and ternary (k_{-2}) opening rates in the presence of complementary nucleotides.

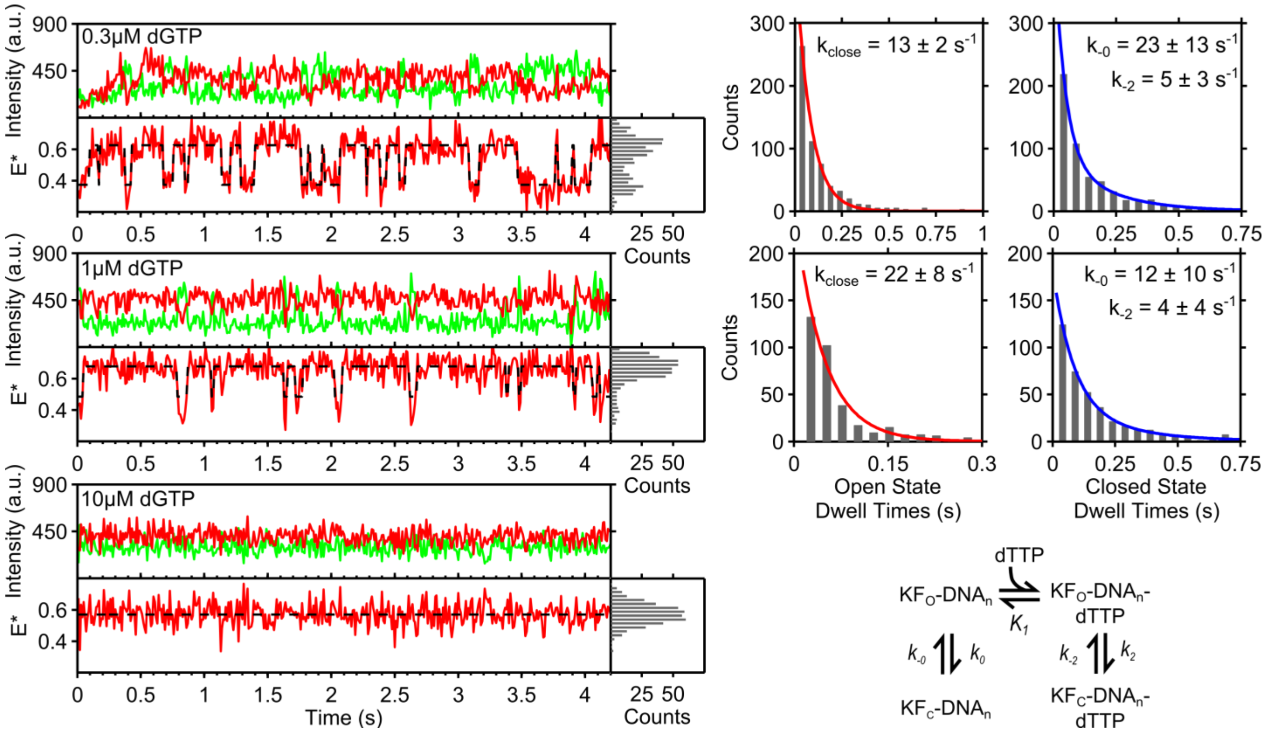

SI Figure 8 Fingers conformations in the presence of complementary nucleotide (C-dGTP).
Data was acquired for 5 min using continuous green excitation at 1.5 mW, with a 10 ms frame time. Fluorescence intensity and E* traces of KF-DNA complexes in the presence of 0.3 µM, 1 µM and 10 µM of complementary nucleotide (C-dGTP). E* histograms are in the right hand panels. Fingers-open and fingers-closed dwell times in the presence of 0.3 µM, 1 µM and 10 µM of dGTP. The open dwells were fit to a single exponential (red) extracting an observed closing rate (k_{close}; see eq.1, main text). The closed dwell times were fit to a double exponential, with a fast and a slow opening rate, representing binary (k_{-0}) and ternary (k_{-2}) complex opening. The reaction scheme seen in Figure 3 is reproduced here for clarity.

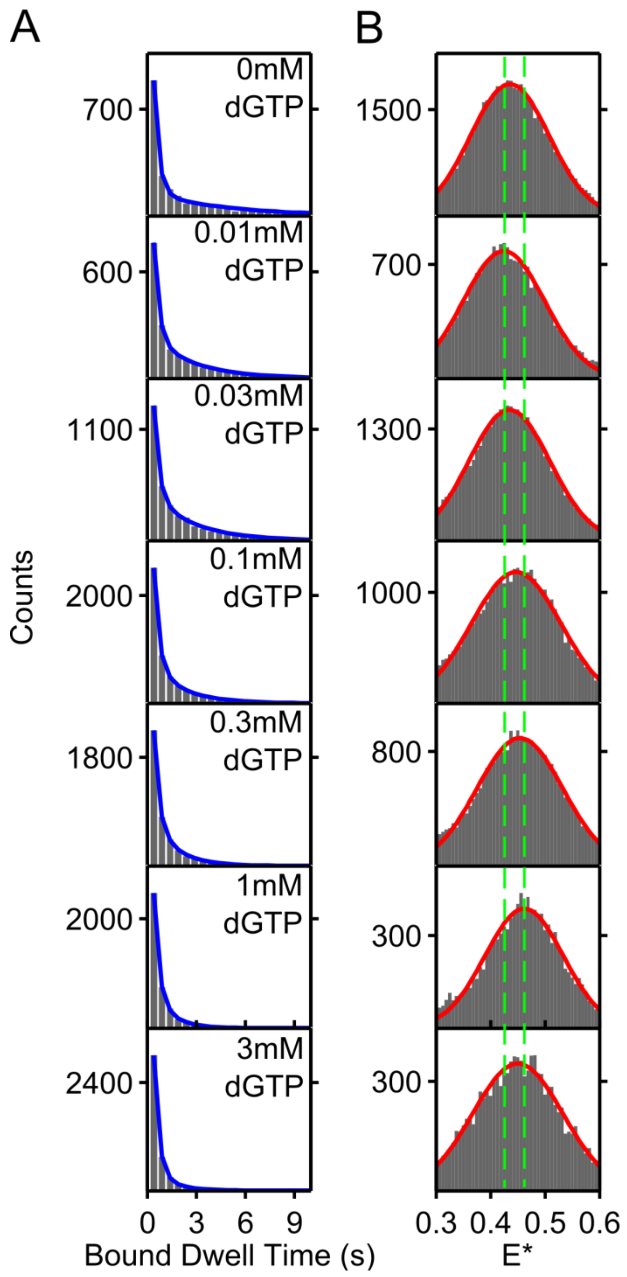

SI Figure 9. **Kinetics of KF dissociation and open state peak position as a function of the concentration of non-complementary nucleotide (A-dGTP).**

A) Dwell time histogram of all KF complexes as a function of mismatched nucleotide concentration. Double exponential fit in blue has the fast dissociation rate ($k_{off,\ fast}$) fixed at 3.1 s^{-1}, and the slow dissociation rate ($k_{off,slow}$) freely fit.

B) Histogram of E* frame values for major population binding events. Peak positions are extracted by fitting a Gaussian profile to histogram values between E^* = 0.3 and E^* = 0.6. Green lines denote the approximate extrema of the peak positions representing the open and the partially closed conformation of the DNA polymerase.

SUPPLEMENTARY INFORMATION

Derivation of the KF fingers-closing rate as a function of complementary nucleotide. We derive the rate of fingers-closing for KF ($k_{obs,\ close}$) as a function of complementary nucleotide, based on the model presented in Fig. 3D, and using a simplified nomenclature. Species C represents the closed binary complex (KF_C-DNA_n); species O represents the open binary complex (KF_O-DNA_n); species ON represents the open ternary complex, with N representing dTTP (KF_O-DNA_n-dTTP); and species CN represents the closed ternary complex (KF_C-DNA_n-dTTP).

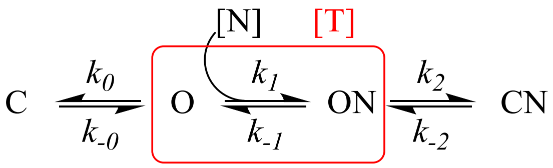

The total concentration of open species, before closing, [T] is given by:

$$[T] = [O] + [ON] \qquad (1)$$

In this treatment, we are considering only the transitions from open states to closed states, since this is the information that is obtained from the single-molecule traces. Since closing can occur from the open binary complex (O), or the open ternary complex (ON); the observed closing rate is the sum of the closing rate from both of these species:

$$Rate\ of\ closing = Rc\ = -(k_0[O] + k_2[ON]) \qquad (2)$$

Assuming a rapid equilibrium for the binding of the nucleotide to the open binary species, we have:

$$[O][N]k_1 = k_{-1}[ON]$$

Therefore: $[ON] = [O][N]K_1$ (3)

Substituting (3) into (1) gives:

$$[T] = [O] + [O][N]K_1$$

$$[O] = \frac{[T]}{1+K_1[N]} \qquad (4)$$

Substituting from (3) into (2) gives:

$$Rc = -(k_0[O] + k_2[O][N]K_1)$$

$$Rc = -[O]\ (k_0 + K_1k_2[N]) \qquad (5)$$

Substituting (4) into (5) gives:

$Rc = -[T]\left\{\frac{k_0+K_1k_2[N]}{1+K_1[N]}\right\}$ thus $k_{obs,\ close} = \frac{k_0+K_1k_2[N]}{1+K_1[N]}$

Rate-limiting step and fidelity. Studies of T7 DNA polymerase suggested that polymerase fidelity may be less dependent on the rate-limiting step than expected (13). Tsai and Johnson observed a conformational change in T7 DNA polymerase upon binding of nucleotide (here we use dTTP for nomenclature consistency with the main text) to the binary complex ($KF_O.DNA_n$), hereby referred to as "fingers-closing". The reaction kinetics measured showed that after nucleotide binding and fingers-closing ($KF_C.DNA_n.dTTP$), nucleotide incorporation is very likely to occur, as the reverse step of fingers-opening (to allow nucleotide release) is much slower than the rate-limiting step in the forwards direction.

$$\mathrm{KF_O \cdot DNA_n} \underset{k_{-1}}{\overset{k_1}{\Leftrightarrow}} \mathrm{KF_O \cdot DNA_n \cdot dTTP} \underset{k_{-2}}{\overset{k_2}{\Leftrightarrow}} \mathrm{KF_C \cdot DNA_n \cdot dTTP} \overset{k_3}{\rightarrow} \mathrm{KF \cdot DNA_{n+1} + PP_i} \quad (1)$$

This was shown mathematically by deriving the catalytic efficiency (k_{cat}/K_m) for the reaction scheme in eq. 1, which becomes independent of the exact rate constant of the rate limiting step when fingers-opening is much slower than the rate-limiting step (since $k_{3,T7\ DNAP} = 360\ s^{-1} >> k_{-2,\ T7\ DNAP} = 1.6\ s^{-1}$):

$$\frac{k_{cat}}{K_m} = \frac{k_1 k_2 k_3}{k_2 k_3 + k_{-1}(k_{-2} + k_3)} \overset{k_3>>k_{-2}}{\rightarrow} \frac{k_1 k_2}{k_2 + k_{-1}} \overset{k_{-1}>>k_2}{\rightarrow} K_1 k_2 \quad (2)$$

Additionally, the catalytic efficiency for a non-complementary simplifies, since incorporation is slow relative to fingers-opening ($k_{-2}>>k_3$, due to misalignment of chemical groups in the fingers-closed state), and on assuming that ground-state binding is a rapid equilibrium step ($k_{-1}k_{-2}>>k_2k_3$); as a result, k_{cat}/K_m reduces to $K'_1 K'_2 k'_3$. The discrimination ratio D (i.e., the ratio of catalytic efficiencies for the complementary and the non-complementary dNTP; see eq. 3 in (37) and Refs. (13, 38, 40)), reduces to:

$$D = \frac{(k_{cat}/K_m)_{correct}}{(k'_{cat}/K'_m)_{incorrect}} = \frac{K_1 k_2}{K'_1 K'_2 k'_3} \quad (3)$$

Thus, in the case of T7 DNA polymerase, the rate-limiting step for complementary nucleotide does not directly contribute towards fidelity.

For KF, we also observe a slow fingers-opening ($k_{-2} = 6.1\ s^{-1}$) relative to the reaction rate-limiting step ($k_3 \approx k_{inc} = 47\ s^{-1}$, which is the mean single-turnover nucleotide incorporation rate across literature values: $k_{inc} = 46.5\ s^{-1}$ for dTTP, $38\ s^{-1}$ for dGTP, $50\ s^{-1}$ for dATP, and $k_{inc} = 53\ s^{-1}$ for dTTP incorporation (26, 29, 30)). Although the rate difference in KF is less pronounced than for T7 DNA polymerase (which may reflect the lower fidelity of KF) (2), the limit K_1k_2 also applies to KF within a factor of $1/(1+ k_{-2}/k_3) = 0.9$, as calculated by evaluating k_{cat}/K_m at the limit of $k_{-1}>>k_2$ (13) and comparing expressions from Eqs. 2 and 4:

$$\frac{k_{cat}}{K_m} = \frac{k_1 k_2 k_3}{k_2 k_3 + k_{-1}(k_{-2} + k_3)} \overset{k_{-1}>>k_2}{\rightarrow} \frac{K_1 k_2}{(1 + k_{-2}/k_3)} \quad (4)$$

The reaction scheme in Eq. 1 ignores a previously inferred slow post-chemisty step (41) which could complicate the analysis by allowing time for the reverse chemical step to occur (k_{-3}). However, Eq. 1 is a good approximation, since pyrophosphorolysis ($0.31\ s^{-1}$) is slow relative to the post-chemistry step ($15\ s^{-1}$) and thus Step 3 is essentially irreversible (41).
